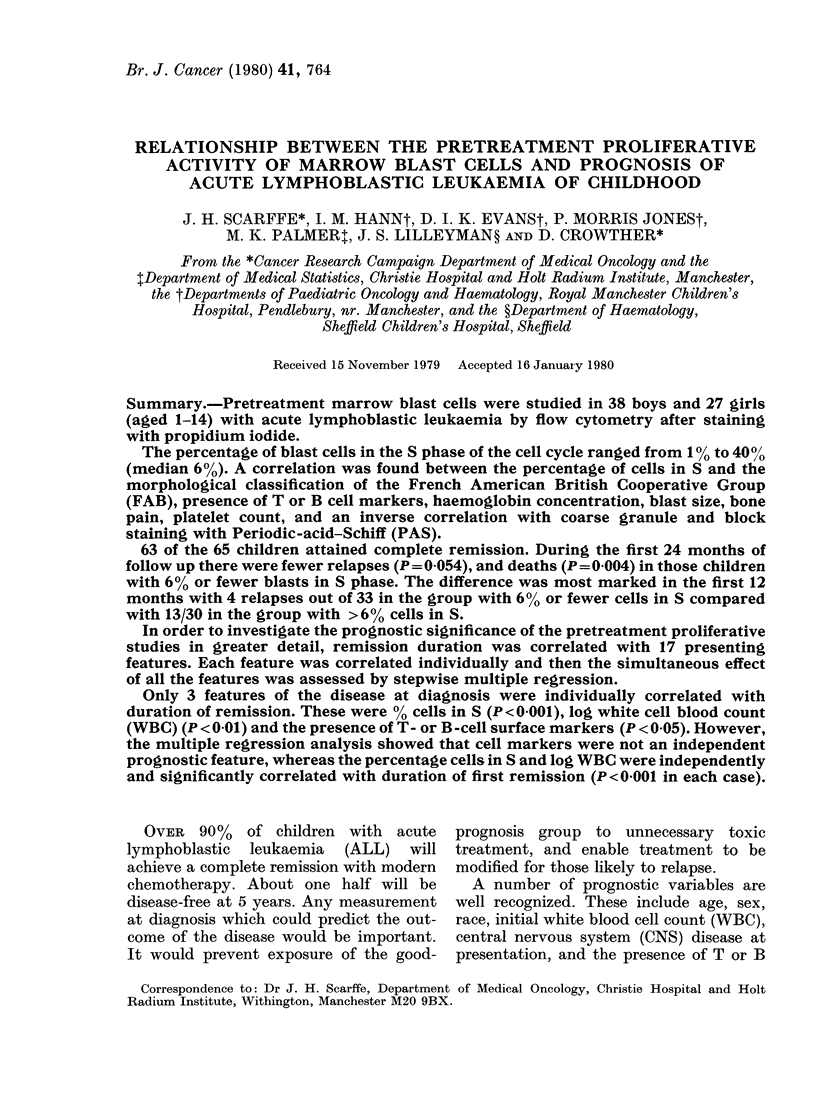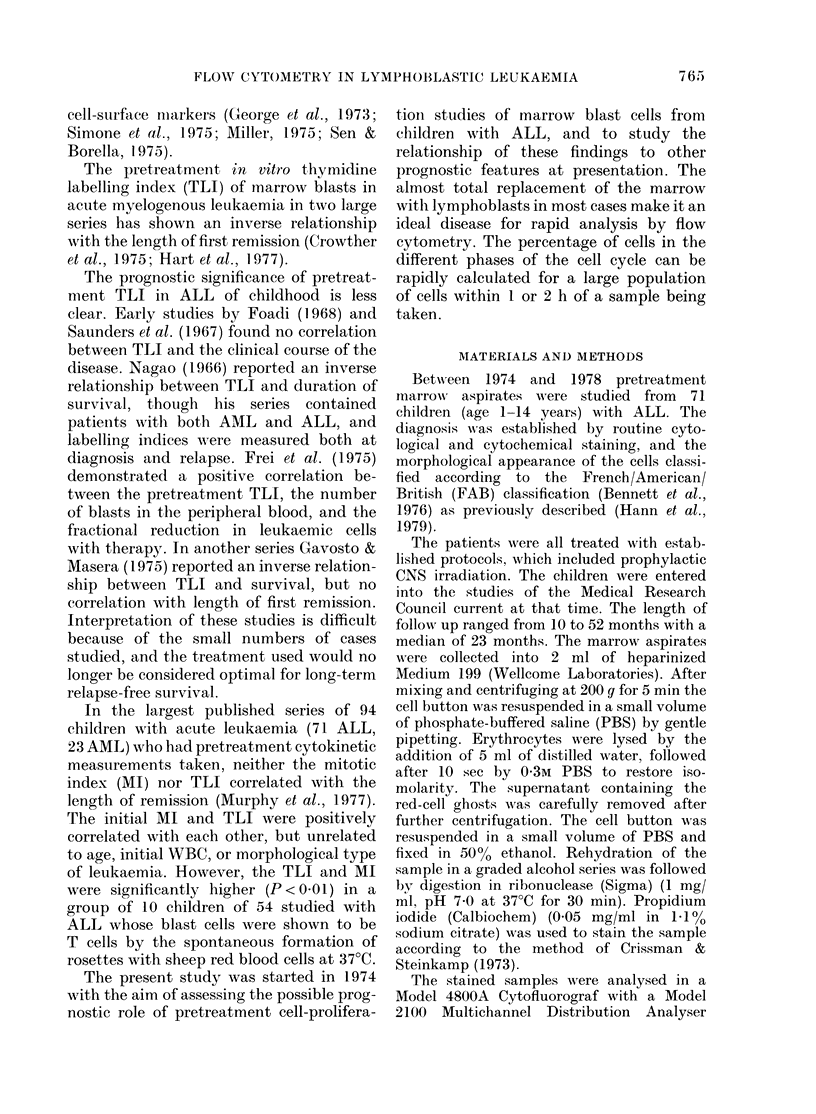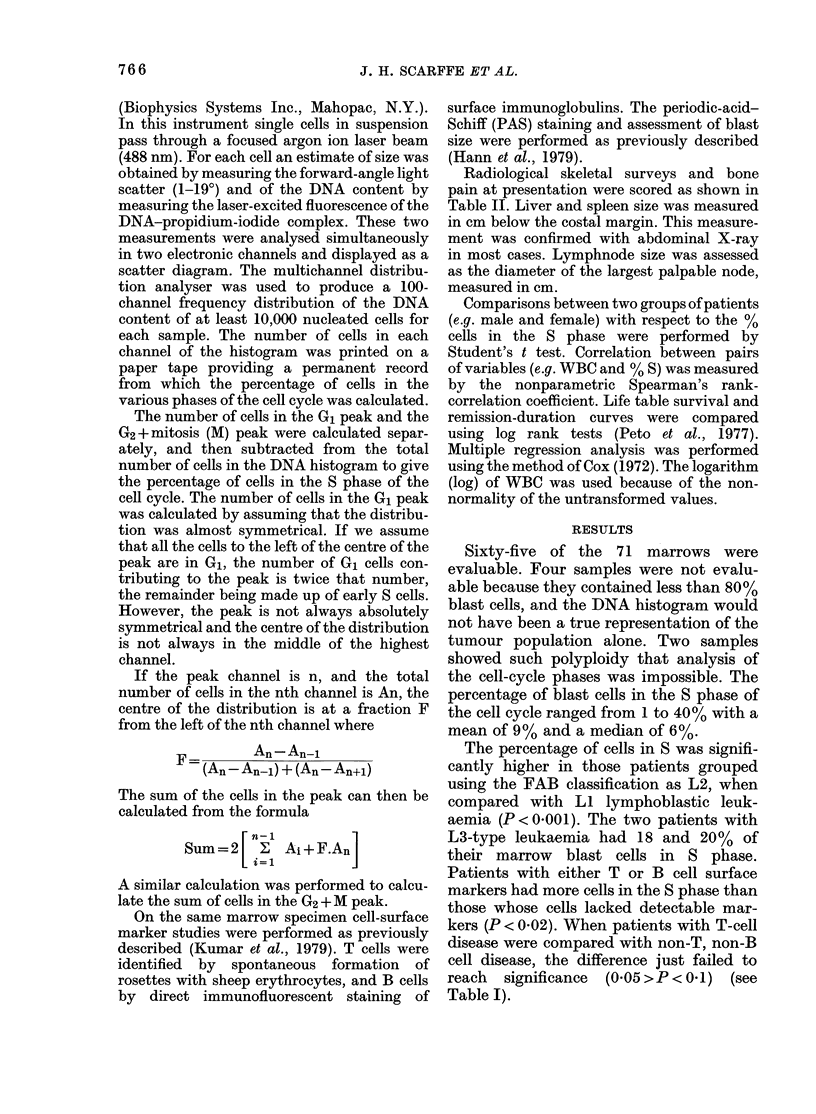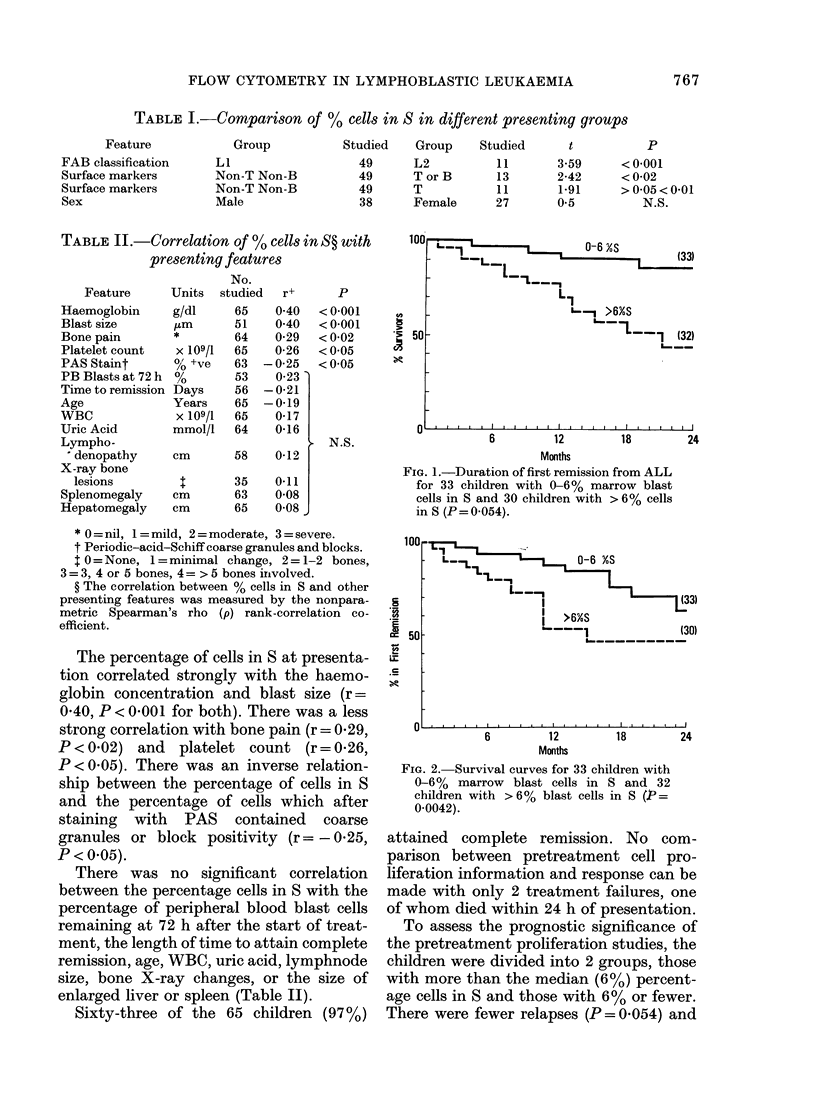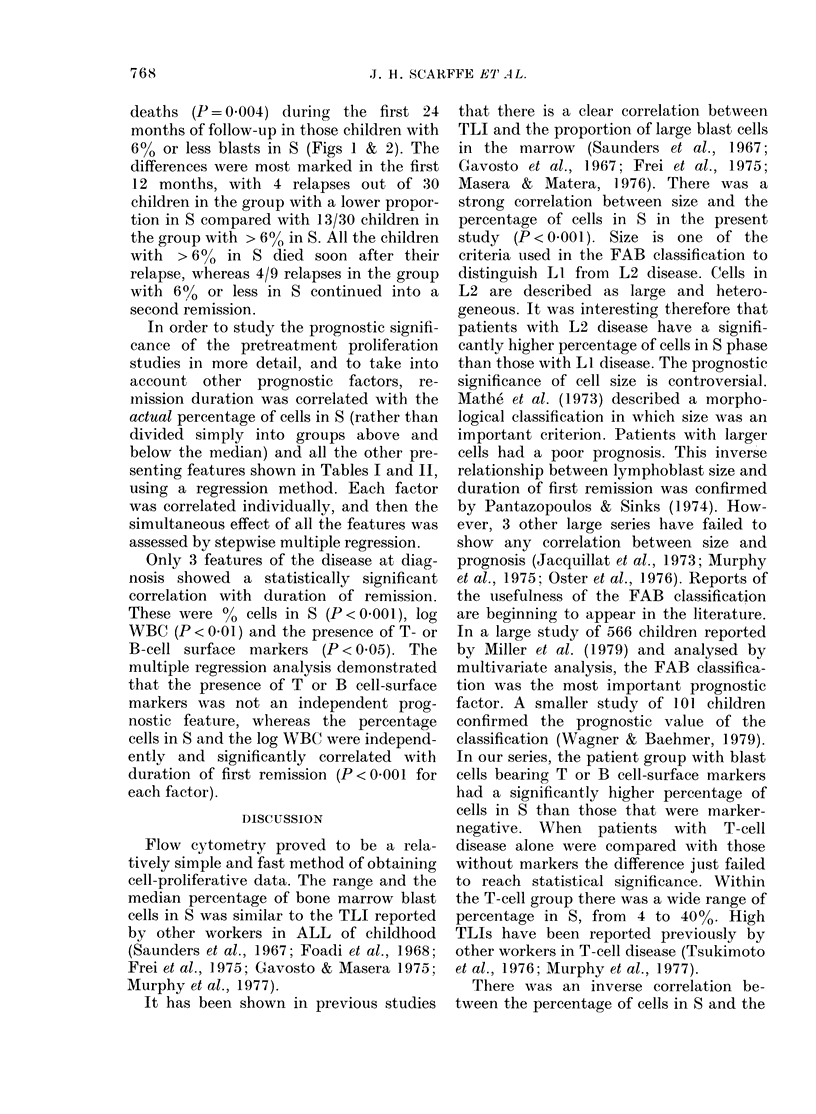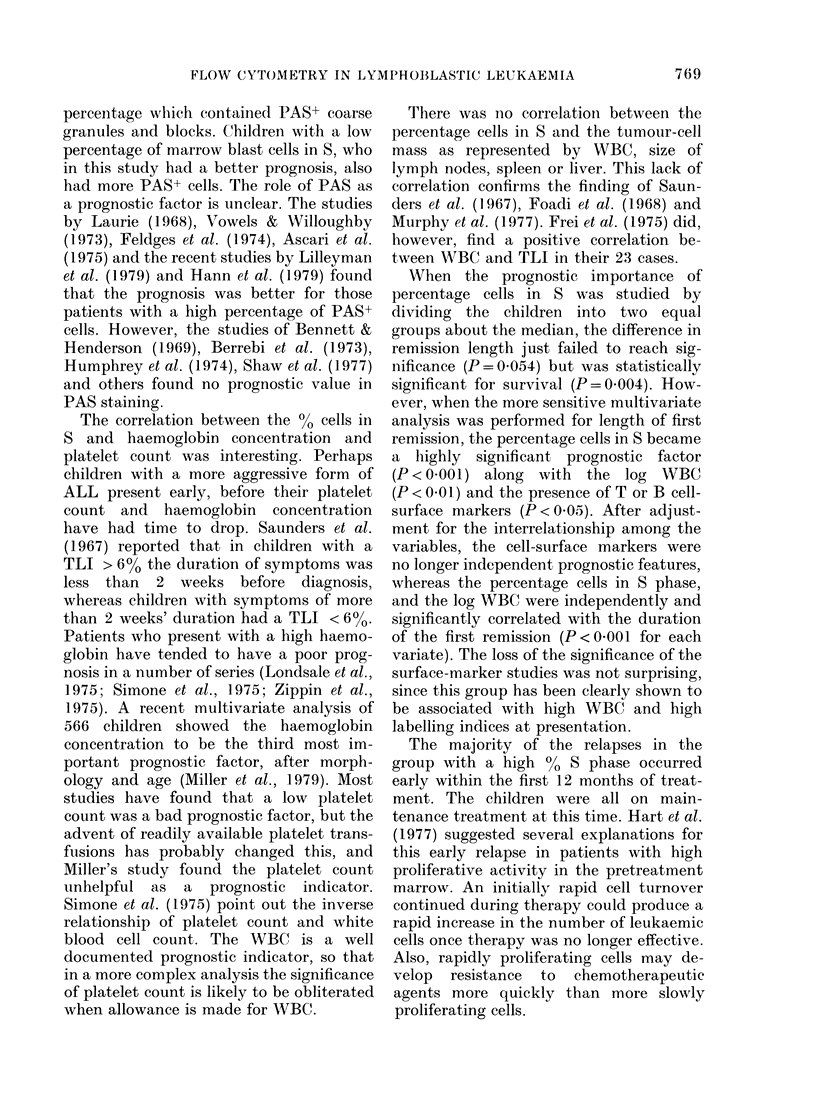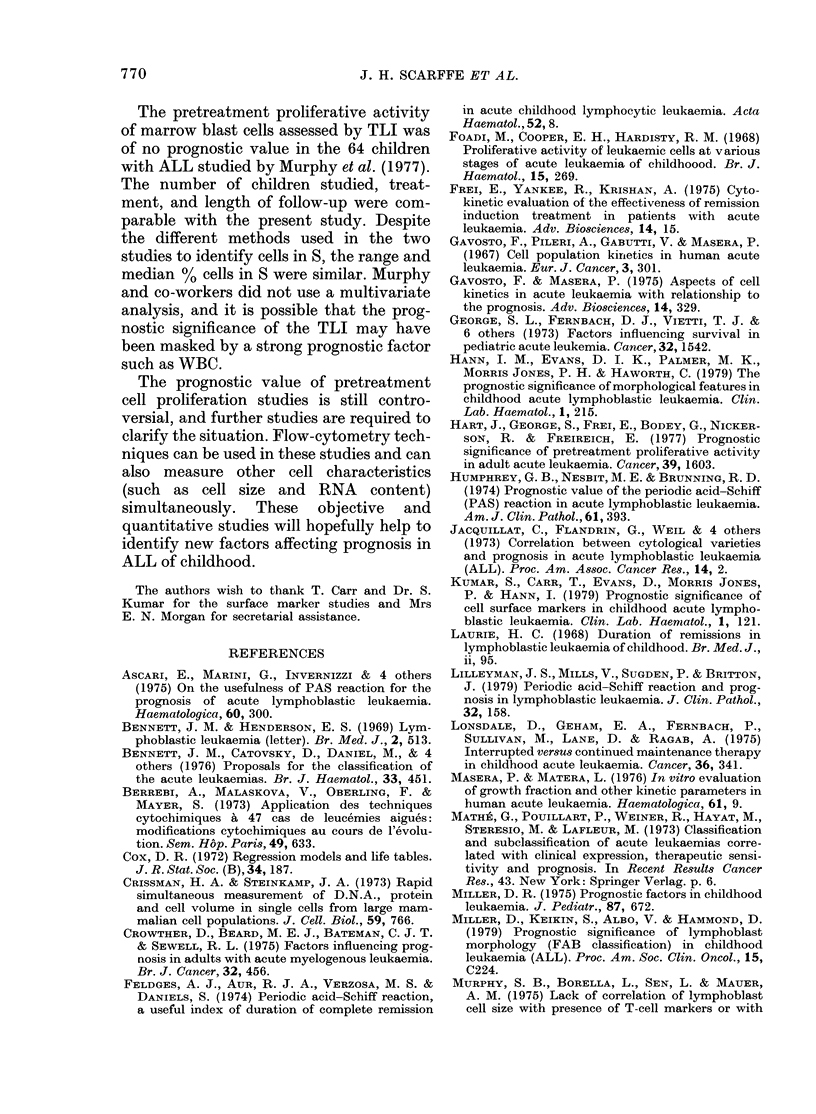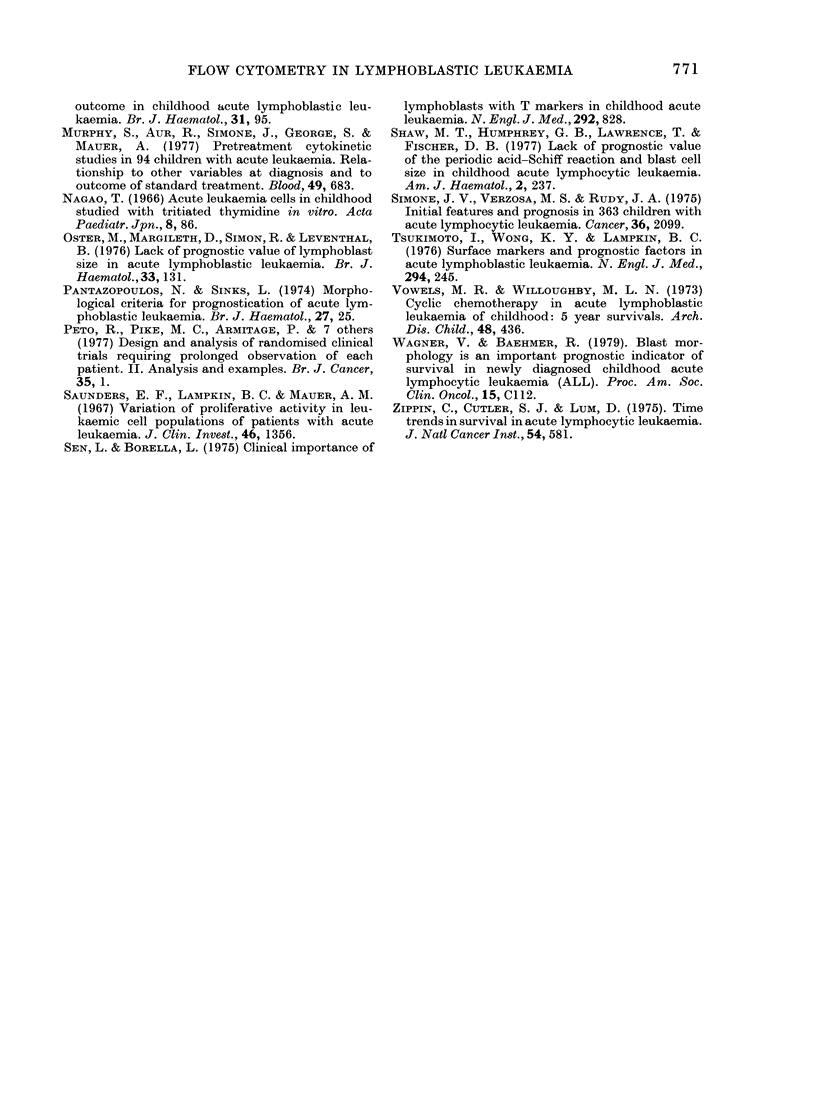# Relationship between the pretreatment proliferative activity of marrow blast cells and prognosis of acute lymphoblastic leukaemia of childhood

**DOI:** 10.1038/bjc.1980.139

**Published:** 1980-05

**Authors:** J. H. Scarffe, I. M. Hann, D. I. K. Evans, P. Morris Jones, M. K. Palmer, J. S. Lilleyman, D. Crowther

## Abstract

Pretreatment marrow blast cells were studied in 38 boys and 27 girls (aged 1-14) with acute lymphoblastic leukaemia by flow cytometry after staining with propidium iodide.

The percentage of blast cells in the S phase of the cell cycle ranged from 1% to 40% (median 6%). A correlation was found between the percentage of cells in S and the morphological classification of the French American British Cooperative Group (FAB), presence of T or B cell markers, haemoglobin concentration, blast size, bone pain, platelet count, and an inverse correlation with coarse granule and block staining with Periodic-acid-Schiff (PAS).

63 of the 65 children attained complete remission. During the first 24 months of follow up there were fewer relapses (*P* = 0·054), and deaths (*P* = 0·004) in those children with 6% or fewer blasts in S phase. The difference was most marked in the first 12 months with 4 relapses out of 33 in the group with 6% or fewer cells in S compared with 13/30 in the group with > 6% cells in S.

In order to investigate the prognostic significance of the pretreatment proliferative studies in greater detail, remission duration was correlated with 17 presenting features. Each feature was correlated individually and then the simultaneous effect of all the features was assessed by stepwise multiple regression.

Only 3 features of the disease at diagnosis were individually correlated with duration of remission. These were% cells in S (*P* < 0·001), log white cell blood count (WBC) (*P* < 0·01) and the presence of T- or B-cell surface markers (*P* < 0·05). However, the multiple regression analysis showed that cell markers were not an independent prognostic feature, whereas the percentage cells in S and log WBC were independently and significantly correlated with duration of first remission (*P* < 0·001 in each case).


*Br. J. Cancer* (1980) **41**, 764

### RELATIONSHIP BETWEEN THE PRETREATMENT PROLIFERATIVE ACTIVITY OF MARROW BLAST CELLS AND PROGNOSIS OF ACUTE LYMPHOBLASTIC LEUKAEMIA OF CHILDHOOD

J. H. SCARFFE*, I. M. HANN†, D. I. K. EVANS†, P. MORRIS JONES†, M. K. PALMER‡, J. S. LILLEYMAN§ AND D. CROWTHER*

*From the *Cancer Research Campaign Department of Medical Oncology and the ‡Department of Medical Statistics, Christie Hospital and Holt Radium Institute, Manchester, the †Departments of Paediatric Oncology and Haematology, Royal Manchester Children's Hospital, Pendlebury, nr. Manchester, and the §Department of Haematology, Sheffield Children's Hospital, Sheffield*

Received 15 November 1979 Accepted 16 January 1980

**Summary.—Pretreatment marrow blast cells were studied in 38 boys and 27 girls (aged 1–14) with acute lymphoblastic leukaemia by flow cytometry after staining with propidium iodide.**

**The percentage of blast cells in the S phase of the cell cycle ranged from 1% to 40% (median 6%). A correlation was found between the percentage of cells in S and the morphological classification of the French American British Cooperative Group (FAB), presence of T or B cell markers, haemoglobin concentration, blast size, bone pain, platelet count, and an inverse correlation with coarse granule and block staining with Periodic-acid–Schiff (PAS).**

**63 of the 65 children attained complete remission. During the first 24 months of follow up there were fewer relapses ($P=0{\cdot}054$), and deaths ($P=0{\cdot}004$) in those children with 6% or fewer blasts in S phase. The difference was most marked in the first 12 months with 4 relapses out of 33 in the group with 6% or fewer cells in S compared with 13/30 in the group with >6% cells in S.**

**In order to investigate the prognostic significance of the pretreatment proliferative studies in greater detail, remission duration was correlated with 17 presenting features. Each feature was correlated individually and then the simultaneous effect of all the features was assessed by stepwise multiple regression.**

**Only 3 features of the disease at diagnosis were individually correlated with duration of remission. These were % cells in S ($P<0{\cdot}001$), log white cell blood count (WBC) ($P<0{\cdot}01$) and the presence of T- or B-cell surface markers ($P<0{\cdot}05$). However, the multiple regression analysis showed that cell markers were not an independent prognostic feature, whereas the percentage cells in S and log WBC were independently and significantly correlated with duration of first remission ($P<0{\cdot}001$ in each case).**

OVER 90% of children with acute lymphoblastic leukaemia (ALL) will achieve a complete remission with modern chemotherapy. About one half will be disease-free at 5 years. Any measurement at diagnosis which could predict the outcome of the disease would be important. It would prevent exposure of the good-prognosis group to unnecessary toxic treatment, and enable treatment to be modified for those likely to relapse.

A number of prognostic variables are well recognized. These include age, sex, race, initial white blood cell count (WBC), central nervous system (CNS) disease at presentation, and the presence of T or B

Correspondence to: Dr J. H. Scarffe, Department of Medical Oncology, Christie Hospital and Holt Radium Institute, Withington, Manchester M20 9BX.

cell-surface markers (George *et al.*, 1973; Simone *et al.*, 1975; Miller, 1975; Sen & Borella, 1975).

The pretreatment *in vitro* thymidine labelling index (TLI) of marrow blasts in acute myelogenous leukaemia in two large series has shown an inverse relationship with the length of first remission (Crowther *et al.*, 1975; Hart *et al.*, 1977).

The prognostic significance of pretreatment TLI in ALL of childhood is less clear. Early studies by Foadi (1968) and Saunders *et al.* (1967) found no correlation between TLI and the clinical course of the disease. Nagao (1966) reported an inverse relationship between TLI and duration of survival, though his series contained patients with both AML and ALL, and labelling indices were measured both at diagnosis and relapse. Frei *et al.* (1975) demonstrated a positive correlation between the pretreatment TLI, the number of blasts in the peripheral blood, and the fractional reduction in leukaemic cells with therapy. In another series Gavosto & Masera (1975) reported an inverse relationship between TLI and survival, but no correlation with length of first remission. Interpretation of these studies is difficult because of the small numbers of cases studied, and the treatment used would no longer be considered optimal for long-term relapse-free survival.

In the largest published series of 94 children with acute leukaemia (71 ALL, 23 AML) who had pretreatment cytokinetic measurements taken, neither the mitotic index (MI) nor TLI correlated with the length of remission (Murphy *et al.*, 1977). The initial MI and TLI were positively correlated with each other, but unrelated to age, initial WBC, or morphological type of leukaemia. However, the TLI and MI were significantly higher ($P < 0{\cdot}01$) in a group of 10 children of 54 studied with ALL whose blast cells were shown to be T cells by the spontaneous formation of rosettes with sheep red blood cells at 37°C.

The present study was started in 1974 with the aim of assessing the possible prognostic role of pretreatment cell-proliferation studies of marrow blast cells from children with ALL, and to study the relationship of these findings to other prognostic features at presentation. The almost total replacement of the marrow with lymphoblasts in most cases make it an ideal disease for rapid analysis by flow cytometry. The percentage of cells in the different phases of the cell cycle can be rapidly calculated for a large population of cells within 1 or 2 h of a sample being taken.

### MATERIALS AND METHODS

Between 1974 and 1978 pretreatment marrow aspirates were studied from 71 children (age 1–14 years) with ALL. The diagnosis was established by routine cytological and cytochemical staining, and the morphological appearance of the cells classified according to the French/American/British (FAB) classification (Bennett *et al.*, 1976) as previously described (Hann *et al.*, 1979).

The patients were all treated with established protocols, which included prophylactic CNS irradiation. The children were entered into the studies of the Medical Research Council current at that time. The length of follow up ranged from 10 to 52 months with a median of 23 months. The marrow aspirates were collected into 2 ml of heparinized Medium 199 (Wellcome Laboratories). After mixing and centrifuging at 200 *g* for 5 min the cell button was resuspended in a small volume of phosphate-buffered saline (PBS) by gentle pipetting. Erythrocytes were lysed by the addition of 5 ml of distilled water, followed after 10 sec by 0·3M PBS to restore isomolarity. The supernatant containing the red-cell ghosts was carefully removed after further centrifugation. The cell button was resuspended in a small volume of PBS and fixed in 50% ethanol. Rehydration of the sample in a graded alcohol series was followed by digestion in ribonuclease (Sigma) (1 mg/ml, pH 7·0 at 37°C for 30 min). Propidium iodide (Calbiochem) (0·05 mg/ml in 1·1% sodium citrate) was used to stain the sample according to the method of Crissman & Steinkamp (1973).

The stained samples were analysed in a Model 4800A Cytofluorograf with a Model 2100 Multichannel Distribution Analyser

(Biophysics Systems Inc., Mahopac, N.Y.). In this instrument single cells in suspension pass through a focused argon ion laser beam (488 nm). For each cell an estimate of size was obtained by measuring the forward-angle light scatter (1–19°) and of the DNA content by measuring the laser-excited fluorescence of the DNA–propidium-iodide complex. These two measurements were analysed simultaneously in two electronic channels and displayed as a scatter diagram. The multichannel distribution analyser was used to produce a 100-channel frequency distribution of the DNA content of at least 10,000 nucleated cells for each sample. The number of cells in each channel of the histogram was printed on a paper tape providing a permanent record from which the percentage of cells in the various phases of the cell cycle was calculated.

The number of cells in the $G_1$ peak and the $G_2$ + mitosis (M) peak were calculated separately, and then subtracted from the total number of cells in the DNA histogram to give the percentage of cells in the S phase of the cell cycle. The number of cells in the $G_1$ peak was calculated by assuming that the distribution was almost symmetrical. If we assume that all the cells to the left of the centre of the peak are in $G_1$, the number of $G_1$ cells contributing to the peak is twice that number, the remainder being made up of early S cells. However, the peak is not always absolutely symmetrical and the centre of the distribution is not always in the middle of the highest channel.

If the peak channel is n, and the total number of cells in the nth channel is An, the centre of the distribution is at a fraction F from the left of the nth channel where

$$F = \frac{A_n - A_{n-1}}{(A_n - A_{n-1}) + (A_n - A_{n+1})}$$

The sum of the cells in the peak can then be calculated from the formula

$$\text{Sum} = 2\left[\sum_{i=1}^{n-1} A_i + F.A_n\right]$$

A similar calculation was performed to calculate the sum of cells in the $G_2$ + M peak.

On the same marrow specimen cell-surface marker studies were performed as previously described (Kumar *et al.*, 1979). T cells were identified by spontaneous formation of rosettes with sheep erythrocytes, and B cells by direct immunofluorescent staining of surface immunoglobulins. The periodic-acid–Schiff (PAS) staining and assessment of blast size were performed as previously described (Hann *et al.*, 1979).

Radiological skeletal surveys and bone pain at presentation were scored as shown in Table II. Liver and spleen size was measured in cm below the costal margin. This measurement was confirmed with abdominal X-ray in most cases. Lymphnode size was assessed as the diameter of the largest palpable node, measured in cm.

Comparisons between two groups of patients (*e.g.* male and female) with respect to the % cells in the S phase were performed by Student's *t* test. Correlation between pairs of variables (*e.g.* WBC and % S) was measured by the nonparametric Spearman's rank-correlation coefficient. Life table survival and remission-duration curves were compared using log rank tests (Peto *et al.*, 1977). Multiple regression analysis was performed using the method of Cox (1972). The logarithm (log) of WBC was used because of the non-normality of the untransformed values.

### RESULTS

Sixty-five of the 71 marrows were evaluable. Four samples were not evaluable because they contained less than 80% blast cells, and the DNA histogram would not have been a true representation of the tumour population alone. Two samples showed such polyploidy that analysis of the cell-cycle phases was impossible. The percentage of blast cells in the S phase of the cell cycle ranged from 1 to 40% with a mean of 9% and a median of 6%.

The percentage of cells in S was significantly higher in those patients grouped using the FAB classification as L2, when compared with L1 lymphoblastic leukaemia ($P < 0{\cdot}001$). The two patients with L3-type leukaemia had 18 and 20% of their marrow blast cells in S phase. Patients with either T or B cell surface markers had more cells in the S phase than those whose cells lacked detectable markers ($P < 0{\cdot}02$). When patients with T-cell disease were compared with non-T, non-B cell disease, the difference just failed to reach significance ($0{\cdot}05 > P < 0{\cdot}1$) (see Table I).

TABLE I.—*Comparison of % cells in S in different presenting groups*

| Feature | Group | Studied | Group | Studied | *t* | *P* |
|---|---|---|---|---|---|---|
| FAB classification | L1 | 49 | L2 | 11 | 3·59 | < 0·001 |
| Surface markers | Non-T Non-B | 49 | T or B | 13 | 2·42 | < 0·02 |
| Surface markers | Non-T Non-B | 49 | T | 11 | 1·91 | > 0·05 < 0·01 |
| Sex | Male | 38 | Female | 27 | 0·5 | N.S. |

TABLE II.—*Correlation of % cells in S§ with presenting features*

| Feature | Units | No. studied | r+ | *P* |
|---|---|---|---|---|
| Haemoglobin | g/dl | 65 | 0·40 | < 0·001 |
| Blast size | μm | 51 | 0·40 | < 0·001 |
| Bone pain | * | 64 | 0·29 | < 0·02 |
| Platelet count | x 10⁹/l | 65 | 0·26 | < 0·05 |
| PAS Stain† | % +ve | 63 | − 0·25 | < 0·05 |
| PB Blasts at 72 h | % | 53 | 0·23 | N.S. |
| Time to remission | Days | 56 | − 0·21 | |
| Age | Years | 65 | − 0·19 | |
| WBC | x 10⁹/l | 65 | 0·17 | |
| Uric Acid | mmol/l | 64 | 0·16 | |
| Lymphadenopathy | cm | 58 | 0·12 | |
| X-ray bone lesions | ‡ | 35 | 0·11 | |
| Splenomegaly | cm | 63 | 0·08 | |
| Hepatomegaly | cm | 65 | 0·08 | |

* 0 = nil, 1 = mild, 2 = moderate, 3 = severe.
† Periodic-acid–Schiff coarse granules and blocks.
‡ 0 = None, 1 = minimal change, 2 = 1–2 bones, 3 = 3, 4 or 5 bones, 4 = > 5 bones involved.
§ The correlation between % cells in S and other presenting features was measured by the nonparametric Spearman's rho (ρ) rank-correlation coefficient.

The percentage of cells in S at presentation correlated strongly with the haemoglobin concentration and blast size (r = 0·40, $P < 0·001$ for both). There was a less strong correlation with bone pain (r = 0·29, $P < 0·02$) and platelet count (r = 0·26, $P < 0·05$). There was an inverse relationship between the percentage of cells in S and the percentage of cells which after staining with PAS contained coarse granules or block positivity (r = − 0·25, $P < 0·05$).

There was no significant correlation between the percentage cells in S with the percentage of peripheral blood blast cells remaining at 72 h after the start of treatment, the length of time to attain complete remission, age, WBC, uric acid, lymphnode size, bone X-ray changes, or the size of enlarged liver or spleen (Table II).

Sixty-three of the 65 children (97%) attained complete remission. No comparison between pretreatment cell proliferation information and response can be made with only 2 treatment failures, one of whom died within 24 h of presentation.

FIG. 1.—Duration of first remission from ALL for 33 children with 0–6% marrow blast cells in S and 30 children with > 6% cells in S ($P = 0·054$).

FIG. 2.—Survival curves for 33 children with 0–6% marrow blast cells in S and 32 children with > 6% blast cells in S ($P = 0·0042$).

To assess the prognostic significance of the pretreatment proliferation studies, the children were divided into 2 groups, those with more than the median (6%) percentage cells in S and those with 6% or fewer. There were fewer relapses ($P = 0·054$) and deaths ($P=0{\cdot}004$) during the first 24 months of follow-up in those children with 6% or less blasts in S (Figs 1 & 2). The differences were most marked in the first 12 months, with 4 relapses out of 30 children in the group with a lower proportion in S compared with 13/30 children in the group with > 6% in S. All the children with > 6% in S died soon after their relapse, whereas 4/9 relapses in the group with 6% or less in S continued into a second remission.

In order to study the prognostic significance of the pretreatment proliferation studies in more detail, and to take into account other prognostic factors, remission duration was correlated with the *actual* percentage of cells in S (rather than divided simply into groups above and below the median) and all the other presenting features shown in Tables I and II, using a regression method. Each factor was correlated individually, and then the simultaneous effect of all the features was assessed by stepwise multiple regression.

Only 3 features of the disease at diagnosis showed a statistically significant correlation with duration of remission. These were % cells in S ($P<0{\cdot}001$), log WBC ($P<0{\cdot}01$) and the presence of T- or B-cell surface markers ($P<0{\cdot}05$). The multiple regression analysis demonstrated that the presence of T or B cell-surface markers was not an independent prognostic feature, whereas the percentage cells in S and the log WBC were independently and significantly correlated with duration of first remission ($P<0{\cdot}001$ for each factor).

### DISCUSSION

Flow cytometry proved to be a relatively simple and fast method of obtaining cell-proliferative data. The range and the median percentage of bone marrow blast cells in S was similar to the TLI reported by other workers in ALL of childhood (Saunders *et al.*, 1967; Foadi *et al.*, 1968; Frei *et al.*, 1975; Gavosto & Masera 1975; Murphy *et al.*, 1977).

It has been shown in previous studies that there is a clear correlation between TLI and the proportion of large blast cells in the marrow (Saunders *et al.*, 1967; Gavosto *et al.*, 1967; Frei *et al.*, 1975; Masera & Matera, 1976). There was a strong correlation between size and the percentage of cells in S in the present study ($P<0{\cdot}001$). Size is one of the criteria used in the FAB classification to distinguish L1 from L2 disease. Cells in L2 are described as large and heterogeneous. It was interesting therefore that patients with L2 disease have a significantly higher percentage of cells in S phase than those with L1 disease. The prognostic significance of cell size is controversial. Mathé *et al.* (1973) described a morphological classification in which size was an important criterion. Patients with larger cells had a poor prognosis. This inverse relationship between lymphoblast size and duration of first remission was confirmed by Pantazopoulos & Sinks (1974). However, 3 other large series have failed to show any correlation between size and prognosis (Jacquillat *et al.*, 1973; Murphy *et al.*, 1975; Oster *et al.*, 1976). Reports of the usefulness of the FAB classification are beginning to appear in the literature. In a large study of 566 children reported by Miller *et al.* (1979) and analysed by multivariate analysis, the FAB classification was the most important prognostic factor. A smaller study of 101 children confirmed the prognostic value of the classification (Wagner & Baehmer, 1979). In our series, the patient group with blast cells bearing T or B cell-surface markers had a significantly higher percentage of cells in S than those that were marker-negative. When patients with T-cell disease alone were compared with those without markers the difference just failed to reach statistical significance. Within the T-cell group there was a wide range of percentage in S, from 4 to 40%. High TLIs have been reported previously by other workers in T-cell disease (Tsukimoto *et al.*, 1976; Murphy *et al.*, 1977).

There was an inverse correlation between the percentage of cells in S and the percentage which contained $PAS^+$ coarse granules and blocks. Children with a low percentage of marrow blast cells in S, who in this study had a better prognosis, also had more $PAS^+$ cells. The role of PAS as a prognostic factor is unclear. The studies by Laurie (1968), Vowels & Willoughby (1973), Feldges *et al.* (1974), Ascari *et al.* (1975) and the recent studies by Lilleyman *et al.* (1979) and Hann *et al.* (1979) found that the prognosis was better for those patients with a high percentage of $PAS^+$ cells. However, the studies of Bennett & Henderson (1969), Berrebi *et al.* (1973), Humphrey *et al.* (1974), Shaw *et al.* (1977) and others found no prognostic value in PAS staining.

The correlation between the % cells in S and haemoglobin concentration and platelet count was interesting. Perhaps children with a more aggressive form of ALL present early, before their platelet count and haemoglobin concentration have had time to drop. Saunders *et al.* (1967) reported that in children with a TLI $>6\%$ the duration of symptoms was less than 2 weeks before diagnosis, whereas children with symptoms of more than 2 weeks' duration had a TLI $<6\%$. Patients who present with a high haemoglobin have tended to have a poor prognosis in a number of series (Londsale *et al.*, 1975; Simone *et al.*, 1975; Zippin *et al.*, 1975). A recent multivariate analysis of 566 children showed the haemoglobin concentration to be the third most important prognostic factor, after morphology and age (Miller *et al.*, 1979). Most studies have found that a low platelet count was a bad prognostic factor, but the advent of readily available platelet transfusions has probably changed this, and Miller's study found the platelet count unhelpful as a prognostic indicator. Simone *et al.* (1975) point out the inverse relationship of platelet count and white blood cell count. The WBC is a well documented prognostic indicator, so that in a more complex analysis the significance of platelet count is likely to be obliterated when allowance is made for WBC.

There was no correlation between the percentage cells in S and the tumour-cell mass as represented by WBC, size of lymph nodes, spleen or liver. This lack of correlation confirms the finding of Saunders *et al.* (1967), Foadi *et al.* (1968) and Murphy *et al.* (1977). Frei *et al.* (1975) did, however, find a positive correlation between WBC and TLI in their 23 cases.

When the prognostic importance of percentage cells in S was studied by dividing the children into two equal groups about the median, the difference in remission length just failed to reach significance ($P=0{\cdot}054$) but was statistically significant for survival ($P=0{\cdot}004$). However, when the more sensitive multivariate analysis was performed for length of first remission, the percentage cells in S became a highly significant prognostic factor ($P<0{\cdot}001$) along with the log WBC ($P<0{\cdot}01$) and the presence of T or B cell-surface markers ($P<0{\cdot}05$). After adjustment for the interrelationship among the variables, the cell-surface markers were no longer independent prognostic features, whereas the percentage cells in S phase, and the log WBC were independently and significantly correlated with the duration of the first remission ($P<0{\cdot}001$ for each variate). The loss of the significance of the surface-marker studies was not surprising, since this group has been clearly shown to be associated with high WBC and high labelling indices at presentation.

The majority of the relapses in the group with a high % S phase occurred early within the first 12 months of treatment. The children were all on maintenance treatment at this time. Hart *et al.* (1977) suggested several explanations for this early relapse in patients with high proliferative activity in the pretreatment marrow. An initially rapid cell turnover continued during therapy could produce a rapid increase in the number of leukaemic cells once therapy was no longer effective. Also, rapidly proliferating cells may develop resistance to chemotherapeutic agents more quickly than more slowly proliferating cells.

The pretreatment proliferative activity of marrow blast cells assessed by TLI was of no prognostic value in the 64 children with ALL studied by Murphy *et al.* (1977). The number of children studied, treatment, and length of follow-up were comparable with the present study. Despite the different methods used in the two studies to identify cells in S, the range and median % cells in S were similar. Murphy and co-workers did not use a multivariate analysis, and it is possible that the prognostic significance of the TLI may have been masked by a strong prognostic factor such as WBC.

The prognostic value of pretreatment cell proliferation studies is still controversial, and further studies are required to clarify the situation. Flow-cytometry techniques can be used in these studies and can also measure other cell characteristics (such as cell size and RNA content) simultaneously. These objective and quantitative studies will hopefully help to identify new factors affecting prognosis in ALL of childhood.

The authors wish to thank T. Carr and Dr. S. Kumar for the surface marker studies and Mrs E. N. Morgan for secretarial assistance.

### REFERENCES

ASCARI, E., MARINI, G., INVERNIZZI & 4 others (1975) On the usefulness of PAS reaction for the prognosis of acute lymphoblastic leukaemia. *Haematologica*, **60**, 300.

BENNETT, J. M. & HENDERSON, E. S. (1969) Lymphoblastic leukaemia (letter). *Br. Med. J.*, **2**, 513.

BENNETT, J. M., CATOVSKY, D., DANIEL, M., & 4 others (1976) Proposals for the classification of the acute leukaemias. *Br. J. Haematol.*, **33**, 451.

BERREBI, A., MALASKOVA, V., OBERLING, F. & MAYER, S. (1973) Application des techniques cytochimiques à 47 cas de leucémies aigués: modifications cytochimiques au cours de l'évolution. *Sem. Hôp. Paris*, **49**, 633.

COX, D. R. (1972) Regression models and life tables. *J. R. Stat. Soc.* (B), **34**, 187.

CRISSMAN, H. A. & STEINKAMP, J. A. (1973) Rapid simultaneous measurement of D.N.A., protein and cell volume in single cells from large mammalian cell populations. *J. Cell. Biol.*, **59**, 766.

CROWTHER, D., BEARD, M. E. J., BATEMAN, C. J. T. & SEWELL, R. L. (1975) Factors influencing prognosis in adults with acute myelogenous leukaemia. *Br. J. Cancer*, **32**, 456.

FELDGES, A. J., AUR, R. J. A., VERZOSA, M. S. & DANIELS, S. (1974) Periodic acid–Schiff reaction, a useful index of duration of complete remission in acute childhood lymphocytic leukaemia. *Acta Haematol.*, **52**, 8.

FOADI, M., COOPER, E. H., HARDISTY, R. M. (1968) Proliferative activity of leukaemic cells at various stages of acute leukaemia of childhoood. *Br. J. Haematol.*, **15**, 269.

FREI, E., YANKEE, R., KRISHAN, A. (1975) Cytokinetic evaluation of the effectiveness of remission induction treatment in patients with acute leukaemia. *Adv. Biosciences*, **14**, 15.

GAVOSTO, F., PILERI, A., GABUTTI, V. & MASERA, P. (1967) Cell population kinetics in human acute leukaemia. *Eur. J. Cancer*, **3**, 301.

GAVOSTO, F. & MASERA, P. (1975) Aspects of cell kinetics in acute leukaemia with relationship to the prognosis. *Adv. Biosciences*, **14**, 329.

GEORGE, S. L., FERNBACH, D. J., VIETTI, T. J. & 6 others (1973) Factors influencing survival in pediatric acute leukemia. *Cancer*, **32**, 1542.

HANN, I. M., EVANS, D. I. K., PALMER, M. K., MORRIS JONES, P. H. & HAWORTH, C. (1979) The prognostic significance of morphological features in childhood acute lymphoblastic leukaemia. *Clin. Lab. Haematol.*, **1**, 215.

HART, J., GEORGE, S., FREI, E., BODEY, G., NICKERSON, R. & FREIREICH, E. (1977) Prognostic significance of pretreatment proliferative activity in adult acute leukaemia. *Cancer*, **39**, 1603.

HUMPHREY, G. B., NESBIT, M. E. & BRUNNING, R. D. (1974) Prognostic value of the periodic acid–Schiff (PAS) reaction in acute lymphoblastic leukaemia. *Am. J. Clin. Pathol.*, **61**, 393.

JACQUILLAT, C., FLANDRIN, G., WEIL & 4 others (1973) Correlation between cytological varieties and prognosis in acute lymphoblastic leukaemia (ALL). *Proc. Am. Assoc. Cancer Res.*, **14**, 2.

KUMAR, S., CARR, T., EVANS, D., MORRIS JONES, P. & HANN, I. (1979) Prognostic significance of cell surface markers in childhood acute lymphoblastic leukaemia. *Clin. Lab. Haematol.*, **1**, 121.

LAURIE, H. C. (1968) Duration of remissions in lymphoblastic leukaemia of childhood. *Br. Med. J.*, ii, 95.

LILLEYMAN, J. S., MILLS, V., SUGDEN, P. & BRITTON, J. (1979) Periodic acid–Schiff reaction and prognosis in lymphoblastic leukaemia. *J. Clin. Pathol.*, **32**, 158.

LONSDALE, D., GEHAM, E. A., FERNBACH, P., SULLIVAN, M., LANE, D. & RAGAB, A. (1975) Interrupted *versus* continued maintenance therapy in childhood acute leukaemia. *Cancer*, **36**, 341.

MASERA, P. & MATERA, L. (1976) *In vitro* evaluation of growth fraction and other kinetic parameters in human acute leukaemia. *Haematologica*, **61**, 9.

MATHÉ, G., POUILLART, P., WEINER, R., HAYAT, M., STERESIO, M. & LAFLEUR, M. (1973) Classification and subclassification of acute leukaemias correlated with clinical expression, therapeutic sensitivity and prognosis. In *Recent Results Cancer Res.*, 43. New York: Springer Verlag. p. 6.

MILLER, D. R. (1975) Prognostic factors in childhood leukaemia. *J. Pediatr.*, **87**, 672.

MILLER, D., KEIKIN, S., ALBO, V. & HAMMOND, D. (1979) Prognostic significance of lymphoblast morphology (FAB classification) in childhood leukaemia (ALL). *Proc. Am. Soc. Clin. Oncol.*, **15**, C224.

MURPHY, S. B., BORELLA, L., SEN, L. & MAUER, A. M. (1975) Lack of correlation of lymphoblast cell size with presence of T-cell markers or with outcome in childhood acute lymphoblastic leukaemia. *Br. J. Haematol.*, **31**, 95.

MURPHY, S., AUR, R., SIMONE, J., GEORGE, S. & MAUER, A. (1977) Pretreatment cytokinetic studies in 94 children with acute leukaemia. Relationship to other variables at diagnosis and to outcome of standard treatment. *Blood*, **49**, 683.

NAGAO, T. (1966) Acute leukaemia cells in childhood studied with tritiated thymidine *in vitro*. *Acta Paediatr. Jpn.*, **8**, 86.

OSTER, M., MARGILETH, D., SIMON, R. & LEVENTHAL, B. (1976) Lack of prognostic value of lymphoblast size in acute lymphoblastic leukaemia. *Br. J. Haematol.*, **33**, 131.

PANTAZOPOULOS, N. & SINKS, L. (1974) Morphological criteria for prognostication of acute lymphoblastic leukaemia. *Br. J. Haematol.*, **27**, 25.

PETO, R., PIKE, M. C., ARMITAGE, P. & 7 others (1977) Design and analysis of randomised clinical trials requiring prolonged observation of each patient. II. Analysis and examples. *Br. J. Cancer*, **35**, 1.

SAUNDERS, E. F., LAMPKIN, B. C. & MAUER, A. M. (1967) Variation of proliferative activity in leukaemic cell populations of patients with acute leukaemia. *J. Clin. Invest.*, **46**, 1356.

SEN, L. & BORELLA, L. (1975) Clinical importance of lymphoblasts with T markers in childhood acute leukaemia. *N. Engl. J. Med.*, **292**, 828.

SHAW, M. T., HUMPHREY, G. B., LAWRENCE, T. & FISCHER, D. B. (1977) Lack of prognostic value of the periodic acid–Schiff reaction and blast cell size in childhood acute lymphocytic leukaemia. *Am. J. Haematol.*, **2**, 237.

SIMONE, J. V., VERZOSA, M. S. & RUDY, J. A. (1975) Initial features and prognosis in 363 children with acute lymphocytic leukaemia. *Cancer*, **36**, 2099.

TSUKIMOTO, I., WONG, K. Y. & LAMPKIN, B. C. (1976) Surface markers and prognostic factors in acute lymphoblastic leukaemia. *N. Engl. J. Med.*, **294**, 245.

VOWELS, M. R. & WILLOUGHBY, M. L. N. (1973) Cyclic chemotherapy in acute lymphoblastic leukaemia of childhood: 5 year survivals. *Arch. Dis. Child.*, **48**, 436.

WAGNER, V. & BAEHMER, R. (1979). Blast morphology is an important prognostic indicator of survival in newly diagnosed childhood acute lymphocytic leukaemia (ALL). *Proc. Am. Soc. Clin. Oncol.*, **15**, C112.

ZIPPIN, C., CUTLER, S. J. & LUM, D. (1975). Time trends in survival in acute lymphocytic leukaemia. *J. Natl Cancer Inst.*, **54**, 581.